# Associations of daytime napping and nighttime sleep quality with depressive symptoms in older Chinese: the Guangzhou biobank cohort study

**DOI:** 10.1186/s12877-023-04579-6

**Published:** 2023-12-19

**Authors:** Weisen Zhang, Baijing Zhou, Chaoqiang Jiang, Yali Jin, Tong Zhu, Feng Zhu, Kar Keung Cheng, Tai Hing Lam, Lin Xu

**Affiliations:** 1 Molecular Epidemiology Research Center, Guangzhou Twelfth People’s Hospital, Guangzhou, 510620 Guangdong China; 2 School of Public Health, Sun Yat-sen University, Guangzhou, China; 3 Institute of Applied Health Research, University of Birmingham, Birmingham, UK; 4 School of Public Health, The University of Hong Kong, Hong Kong, China

**Keywords:** Depressive symptoms, Daytime napping, Nighttime sleep quality, Older people, Guangzhou biobank cohort study

## Abstract

**Background:**

Poor sleep quality has been linked to depression in older adults, but results of the association between daytime napping and depression remains limited and conflicting. Moreover, whether the association of daytime napping with depression varies by nighttime sleep quality is unclear. Hence, we examined the associations of daytime napping and nighttime sleep quality with depressive symptoms in older Chinese.

**Methods:**

A total of 16,786 participants aged ≥50 from the Guangzhou Biobank Cohort Study second-round examination (2008–2012) were included in this cross-sectional study. Geriatric Depression Scale (GDS-15), Pittsburgh Sleep Quality Index (PSQI), napping and demographic data were collected by face-to-face interview using a computerized questionnaire. Logistic regression was used to calculate odds ratio (OR) of depressive symptoms for napping and sleep quality.

**Results:**

The prevalence of depressive symptoms (GDS score > 5) and poor global sleep quality (PSQI score ≥ 6) was 5.3 and 31.9%, respectively. Compared to non-nappers, nappers showed significantly higher odds of depressive symptoms, with OR (95% confidence interval (CI)) being 1.28 (1.11–1.49). The odds of depressive symptoms for daytime napping varied by nighttime sleep quality (P for interaction = 0.04). In good-quality sleepers, compared to non-nappers, nappers had significantly higher odds of depressive symptoms, with OR (95% CI) being 1.57 (1.23–2.01), whereas no association was found in poor-quality sleepers (OR = 1.13, 0.94–1.36).

**Conclusion:**

Napping was associated with higher odds of depressive symptoms in older people, and the association was stronger in good-quality sleepers.

**Supplementary Information:**

The online version contains supplementary material available at 10.1186/s12877-023-04579-6.

## Introduction

Depression is an important public health problem, causing the highest burden of non-fatal disease worldwide [1]. An estimation from the World Health Organization showed that 322 million people suffer from depression [2]. The prevalence of depression is about 4.7% globally, which was higher in the ageing population (8–16%) [3]. In a recent cross-sectional study of 18,944 Chinese adults, the prevalence of depressive symptoms was showed as high as 16.3% [4].

Sleep is one of the most important lifestyle factors. Previous meta-analysis of 23 prospective studies showed that older adults with persistent sleep disturbances had higher risk of the development, recurrence, and worsening of depression [5]. A recent cross-sectional study of 0.5 million Chinese adults also showed that sleep disturbances were associated with depression [6]. Also, other abnormal nighttime sleep behaviors, such as extended nighttime sleep duration, and poor sleep quality, were also associated with depressive symptoms [7, 8]. Poor sleep quality was associated with higher odds of depressive symptoms [8–11], showing a J-shaped relationship between the Pittsburgh Sleep Quality Index (PSQI) scores and depressive symptoms [12]. In addition, only two previous cross-sectional studies reported positive combined association between sleep quality and nighttime sleep duration on depressive symptoms [12, 13]. One on 28,202 Chinese rural adults showed that poor-quality sleepers with long nighttime duration (≥10 h/day) had the highest odds of depressive symptoms [12]. The other one on 6126 Singapore adults also showed that poor-quality sleepers with long nighttime duration (≥9 h/day) had the highest odds of major depressive disorder [13]. None of them, however, considered the association of daytime napping with depressive symptoms.

In China, daytime napping is traditionally considered as a part of healthy life, and also an adjunct to the usual nocturnal sleep period [14]. Napping was associated with higher risks of chronic diseases in previous studies, such as hypertension, diabetes, cardiovascular disease, metabolic syndrome, and cancer [14–17]. Previous papers in the Guangzhou Biobank Cohort Study (GBCS) also reported that frequent napping was positively associated with diabetes and impaired fasting glucose [18], and cognitive impairment in older Chinese [19, 20]. However, the association between daytime napping and depressive symptoms is less clear. Some cross-sectional studies showed positive associations of daytime napping with depressive symptoms [21] and depression [15, 22], while the other one found no significant association [23]. Moreover, a study found that napping was associated with better mood and less depressive symptoms [24]. Thus, whether napping plays a positive, negative or null role in depressive symptoms and whether the associations, if any, are independent of, or modified by nighttime sleep quality are unclear.

Using data from the Guangzhou Biobank cohort study (GBCS), we examined whether depressive symptoms were associated with daytime napping and nighttime sleep quality separately and combined. We hypothesized that both daytime napping (simplified as “napping”) and nighttime sleep quality (simplified as “sleep quality”) were associated with depressive symptoms, and the association of napping with depressive symptoms was modified by sleep quality.

## Methods

### Participants

The Guangzhou Biobank Cohort Study (GBCS) is a three-way collaborative project of the Guangzhou Twelfth People’s Hospital, the University of Hong Kong, and the University of Birmingham. Further information of the GBCS has been reported elsewhere [25]. All GBCS participants were recruited from members of a community social and welfare association, which is open to permanent Guangzhou residents aged 50 years or above. A total of 18,129 participants returned for the second-round examination. After excluding the missing data on sleeping-related factors or the assessment of depressive symptoms, 16,786 participants (73% women) with all variables of interests were included in this cross-sectional study. Their mean age was 65.2 (standard deviation = 7.1) years (Table 1). The study received approval from the Medical Ethics Committee of the Guangzhou Medical Association, and all participants gave written informed consent. Data were collected by face-to-face interview using a computerized questionnaire.

**Table 1:** Characteristics of 16,786 participants by depressive symptoms group

Variables	GDS-15 score ≤ 5 (*N* = 15,897)	GDS-15 score > 5 (*N* = 889)	*P* values
Age (years), mean (SD)	65.1 (7.3)	67.2 (7.0)	< 0.001
Sex, women, %	11,585 (72.9)	660 (74.2)	0.37
BMI (kg/m^2^), mean (SD)	23.8 (3.5)	23.8 (3.9)	0.95
Education level, %			
Primary or below	6038 (38.0)	478 (53.8)	
Middle school	8371 (52.6)	369 (41.5)	< 0.001
College and above	1488 (9.4)	42 (4.7)	
Occupation, %			
Manual	7298 (45.9)	561 (63.1)	
Non-manual	5496 (34.6)	218 (24.5)	< 0.001
Other	3103 (19.5)	110 (12.4)	
Family income (Yuan/ year), %			
< 10,000	1863 (11.7)	172 (19.3)	
10,000–19,999	2384 (15.0)	268 (30.1)	< 0.001
≥20,000	11,650 (73.3)	449 (50.6)	
Physical activity, %			
No	118 (0.7)	19 (2.1)	
Low level (walking)	7603 (47.8)	517 (58.2)	< 0.001
Medium level	7606 (47.8)	334 (37.6)	
High level	570 (3.6)	19 (2.1)	
Smoking status, %			
Never	12,589 (79.2)	698 (78.5)	
Former	1923 (12.1)	124 (13.9)	0.15
Current	1385 (8.7)	67 (7.5)	
Alcohol consumption, %			
Never	7528 (47.4)	398 (44.7)	
Former	42 (0.3)	9 (1.0)	< 0.001
Current	8327 (52.4)	482 (54.3)	
Support from family members, % positive	15,709 (98.8)	850 (95.6)	< 0.001
Contacts with relatives and friends, %			
Not at all	351 (2.2)	61 (6.9)	
<once/month	2410 (15.2)	193 (21.7)	
≥once/month	6044 (38.0)	280 (31.5)	< 0.001
≥once/week	5472 (34.4)	256 (28.9)	
Almost daily	1620 (10.2)	99 (11.1)	
Presence of chronic diseases, %			
Yes	9595 (60.4)	614 (69.0)	< 0.001
Self-rated health, % good	2759 (17.4)	122 (13.7)	0.005
Daytime napping, % nappers	9508 (59.8)	579 (65.1)	< 0.001
Global sleep quality, %			
Poor (PSQI ≥6)	4785 (30.1)	571 (64.2)	< 0.001

Data are mean (SD) or number (percentage, %). *BMI* body mass index, *PSQI* Pittsburgh Sleep Quality Index. 1 USD = 7 Yuan

Chronic diseases including hypertension, diabetes, cardiovascular disease, COPD and cancer

*COPD* chronic obstructive pulmonary diseases. COPD was defined as forced expiratory volume in 1 second (FEV1)/ forced vital capacity (FVC) of less than 0·70, but without the use of a bronchodilator [26]

### Assessment of depressive symptoms

The presence of depressive symptoms was assessed by the shortened 15-item Geriatric Depression Scale (GDS-15, score 0–15), a self-report instrument used extensively for comprehensive geriatric assessment of depressive symptoms, and with higher scores indicating more severity of depressive symptoms [27]. Reliability of the Chinese version of GDS-15 has been reported in previous studies, with Cronbach’s α coefficients ranging from 0.75 to 0.85, and test-retest reliability ranging from 0.68 to 0.83 [28–31]. Also, the GDS-15 has been widely used in older Chinese [32–35], with cutoffs for depressive symptoms ranging from 4 to 8 [33–36], we used a GDS score of 5 or more as a diagnosis of depressive symptoms, because it was reported to have higher sensitivity (96.3%) and specificity (87.5%) than other cut-off points [34].

### Daytime napping (napping)

According to a previous report from GBCS, participants were required to describe their napping habits by answering the following question: “Did you have a habit of taking daytime naps?” Those reported “no” were categorized into group of non-nappers, and others reported “yes” were categorized into group of nappers [18]. For nappers, the questions related to frequency and duration of napping were asked: “How many times did you take a nap during a usual week?” and “On overage, how long did you spend for napping each day?”. Moreover, to facilitate comparison to previous papers, the respondents were categorized into 1–3 days/week, 4–6 days/week and daily for napping frequency [19, 20] and < 60 min/day, 60–90 min/day and > 90 min/day for napping duration [24, 37], respectively.

### Nighttime sleep quality (sleep quality)

Participants’ quality of sleep at night within the past month was assessed using the Pittsburgh Sleep Quality Index (PSQI) Scale [38] by self-report, which had good reliability and validity, with Cronbach’s α coefficients ranging from 0.73 to 0.83, and test-retest reliability ranging from 0,82 to 0.85. The PSQI scale has been widely used in Chinese populations [39, 40]. PSQI has 19 self-administered items in seven components: subjective sleep quality, sleep latency, sleep duration, habitual sleep efficiency, sleep disturbances, use of sleep medications, and daytime dysfunction. Each of the above seven components scored from 0 and 3, and finally, these components were summed to obtain a global PSQI score (ranging from 0 to 21). This method had been reported in our previous GBCS paper [41]. As a PSQI score greater than 5 yielded a diagnostic sensitivity of 89.6% and specificity of 86.5% (Kappa = 0.75, *P* < 0.001) in distinguishing good-quality and poor-quality sleepers [38], we defined poor global sleep quality by PSQI≥6. We categorized the seven components of PSQI according to the original scoring protocol and previous GBCS papers, including subjective sleep quality (good and poor), sleep latency (≤30 min, 31–60 min, and > 60 min), sleep duration (< 5 h/day, 5–6 h/day, 7–8 h/day, and > 8 h/day), habitual sleep efficiency (≥85%, 75–84%, 65–74, and < 65%), sleep disturbances (not at all/<once per week, and at least once per week), use of sleep medications (not at all/<once per week, and at least once per week), and daytime dysfunction (not at all/<once per week, and at least once per week) [38, 41].

### Potential confounders

Socioeconomic and demographic data were collected, including sex, age (years), education (primary school and below, middle school, or college and above), personal income (< 10,000 Yuan/year, 10,000–19,999 Yuan/year, or ≥ 20,000 Yuan/year, US$1 = 7 Yuan), and occupation (manual workers, non-manual workers, or others). Status of smoking and alcohol consumption was classified as “never, former, and current”. Body Mass Index was calculated by the height and weight of the participants from a physical examination (BMI = weight/height square, kg/m^2^) [18]. Physical activity was categorized into four groups (none/ walking/ medium level/ high level) using the previously validated short-version International Physical Activity Questionnaire [42]. According to previous studies, social isolation was associated with both poor sleep quality [43–45] and depression [46–48], indicating that social isolation might be a potential confounding factor. Therefore, social isolation was included as one of co-variates. Information of social isolation was also collected by self-report, such as getting support from family members and frequency of contacts with relatives or friends. Support from family was assessed by asking participants the following question: “do you think you are getting support from family relationship?” And the answers were categorized into negative and positive. The contact with relatives and friends was part of “non-face-to-face contacts (by telephone/mail)” from the Berkman-Syme Social Network Index (SNI), which has been validated in Chinese population [49]. We additionally included mail as another way of non-face-to-face contact besides telephone, since both telephone and mail were common non-face-to-face contact ways in 2003, before smartphone, Internet, and social media had become popular [50, 51]. According to the previous GBCS papers, the contact with relatives and friends were categorized into not at all, <once/month, ≥once/month, ≥once/week, or almost daily [50–52]. Other data included subjective health status as good or poor, and history of chronic diseases (including hypertension, diabetes, cancer, chronic obstructive pulmonary disease, and coronary heart disease) based on physical examination and self-reported previous physician diagnoses or medication history, which was categorized into none and yes (one or more). In addition, sleep apnea was defined as self-reported stop breathing during sleep or physician-diagnosed apnea. We did not adjust for nocturnal or shift work occupation because almost all participants (99.6%) were retired at the time of survey.

### Statistical analysis

Continuous variables were described as means (standard deviation, SD) and categorical variables as number (percentage, %). For continuous variables, the Kruskal-Wallis T test was used to determine the differences between groups with or without depressive symptoms, and for categorical variables, Pearson chi-square test was used. Binary logistic regression was used to analyze the associations of napping and sleep quality (including the above-mentioned seven components) with depressive symptoms, with adjustment for potential confounders, giving odds ratios (ORs) and 95% confidence intervals (CIs). To provide a clearer understanding of the impact of each type of covariate on the magnitude of associations, we conducted hierarchical regression models to adjust for the covariates in a stepwise manner. Specifically, models 1 to 5 were adjusted for the following set of covariates using hierarchical modeling approach: models by adjusting for demographic variables (age, sex, education level, occupation, and personal income), health behaviors (smoking status, alcohol consumption, and physical activity), social isolation (support from family members, and contacts with relatives and friends), health-related factors (BMI, presence of chronic diseases, and self-rated health). In addition, we also tested the interaction between napping and sleep quality. In order to account for the potential inflation of Type I error due to multiple testing, we applied the Bonferroni correction. Specifically, the original significance level (e.g., α = 0.05) was divided by the number of tests conducted. Given that we performed 11 tests [11 sleep-related exposures in total: three daytime napping (napping, napping frequency, and napping duration) and eight nighttime sleep quality (global sleep quality and seven components)], the adjusted significance level was set at *P* < 0.01, e.g., 0.05/11 [test number] ≈ 0.0045. All data analysis was done using SPSS 26.0 (SPSS, Chicago, IL, USA). A two-sided *P* < 0.05 was considered statistically significant.

## Results

### Characteristics of participants

Of 16,786 participants, 889 (5.3%) had depressive symptoms. Table 1 shows that, compared to those without depressive symptoms, participants with depressive symptoms were older, had lower education level and family income, more manual workers, more frequent alcohol consumption, and less support from family members, less contact with friends and relatives and poorer self-rated health (all *P* < 0.001). Participants with depressive symptoms also had higher prevalence of poor sleep quality, daily naps, and common chronic diseases (all P < 0.001).

Association of napping frequency and duration, and sleep quality with depressive symptoms.

Table 2 shows that, compared to non-nappers, after adjustment for sex, age, BMI, education, occupation, personal income, smoking, alcohol consumption, physical activity, support from family members, contacts with relatives and friends, presence of chronic diseases, self-rated health and sleep quality (Model3), participants with daily napping, napping duration of 60–90 min/day and > 90 min/day had higher odds of depressive symptoms, with OR (95% CI) of 1.38 (1.17–1.62), 1.24 (1.05–1.47) and 1.68 (1.30–2.18), respectively. Additionally, after adjustment for potential confounders and napping (Model 3) those with poor global sleep quality, poor subjective sleep quality, sleep disturbances, sleep medications, or daytime dysfunction at least once per week, had higher odds of depressive symptoms, with OR (95% CI) of 3.56 (3.08–4.12), 2.98 (2.56–3.46), 6.32 (4.92–8.10), 2.92 (2.12–4.02), and 6.42 (5.45–7.55), respectively. The odds of depressive symptoms increased with sleep latency and decreased with sleep duration and sleep efficiency (all P for trend < 0.001). Compared to sleep latency ≤30 min, sleep latency > 60 min showed the highest odds ratios of depressive symptoms, with OR (95% CI) of 3.02 (2.34–3.89), and the OR (95% CI) was 2.32 (1.81–2.97) for sleep duration of < 5 h versus 7-8 h, and 1.94 (1.57–2.40) for sleep efficiency of < 65% versus ≥85%. Upon applying the Bonferroni correction, the association between daytime napping and nighttime sleep quality and depressive symptoms remained statistically significant with a corrected *p*-value of 0.01.

**Table 2:** Prevalence and adjusted OR (95% CI) of depressive symptoms by groups of napping frequency and duration, and Pittsburgh Sleep Quality Index components and global sleep quality

Variables	Depressive symptoms (%)	Model 1	Model 2	Model 3
Napping frequency				
Non-nappers	6699 (4.6)	1.00	1.00	1.00
1–3 days/week	2306 (4.8)	1.13 (0.90, 1.41)	1.11 (0.89, 1.40)	1.11 (0.88, 1.40)
4–6 days/week	1500 (4.6)	1.14 (0.87, 1.50)	1.12 (0.85, 1.47)	1.10 (0.83, 1.45)
daily	6281 (6.4)	1.41 (1.20, 1.65)***	1.38 (1.18, 1.62)***	1.38 (1.17, 1.62)***
P for trend		< 0.001	< 0.001	< 0.001
Napping duration				
Non-nappers	6699 (4.6)	1.00	1.00	1.00
< 60 min	3311 (5.4)	1.24 (1.02, 1.51)*	1.23 (1.01, 1.49)*	1.22 (1.01, 1.49)*
60–90 min	5561 (5.5)	1.26 (1.08, 1.49)**	1.23 (1.04, 1.46)*	1.24 (1.03, 1.45)*
> 90 min	1093 (8.0)	1.72 (1.33, 2.21)***	1.67 (1.30, 2.16)***	1.68 (1.30, 2.18)***
P for trend		< 0.001	< 0.001	< 0.001
Subjective sleep quality				
Good	14,213 (4.1)	1.00	1.00	1.00
Poor	2575(12.2)	3.02 (2.60, 3.50)***	2.97 (2.55, 2.45)***	2.98 (2,57, 3.47)***
P		< 0.001	< 0.001	< 0.001
Sleep latency				
≤30 min	14,544 (4.5)	1.00	1.00	1.00
31–60 min	1670 (8.6)	1.74 (1.43, 2.11)***	1.71 (1.41, 2.08)***	1.70 (1.40, 2.06)***
> 60 min	574 (14.6)	3.03 (2.35, 3.90)***	3.02 (2.34, 3.89)***	3.01 (2.33, 3.88)***
P for trend		< 0.001	< 0.001	< 0.001
Sleep duration				
< 5 h/day	893 (11.0)	2.32 (1.81, 2.97)***	2.28 (1.78, 2.92)***	2.31 (1.80, 2.96)***
5–6 h/day	6503 (6.7)	1.58 (1.36, 1.84)***	1.59 (1.36, 1.85)***	1.59 (1.36, 1.85)***
7–8 h/day	7917 (4.0)	1.00	1.00	1.00
> 8 h/day	843 (2.7)	0.67 (0.43, 1.03)	0.66 (0.43, 1.03)	0.67 (0.44, 1.04)
P for trend		< 0.001	< 0.001	< 0.001
Sleep efficiency				
≥85%	11,348 (4.1)	1.00	1.00	1.00
75–84%	2664 (6.9)	1.54 (1.29, 1.85)***	1.54 (1.29, 1.85)***	1.54 (1.28, 1.85)***
65–74%	1403 (8.3)	1.88 (1.51, 2.34)***	1.85 (1.49, 2.29)***	1.86 (1.50, 2.31)***
< 65%	1373 (9.3)	1.98 (1.60, 2.45)***	1.94 (1.57, 2.40)***	1.94 (1.57, 2.39)***
P for trend		< 0.001	< 0.001	< 0.001
Sleep disturbances				
Not at all/<once per week	16,416 (4.7)	1.00	1.00	1.00
At least once per week	372 (29.8)	6.51 (5.08, 8.34)***	6.41 (5.00, 8.21)***	6.35 (4.95, 8.14)***
P		< 0.001	< 0.001	< 0.001
Sleep medications				
Not at all/<once per week	16,415 (5.1)	1.00	1.00	1.00
At least once per week	373 (13.4)	2.99 (2.18, 4.12)***	2.88 (2.09, 3.96)***	2.91 (2.12, 4.01)***
P		< 0.001	< 0.001	< 0.001
Daytime dysfunction				
Not at all/<once per week	15,576 (3.9)	1.00	1.00	1.00
At least once per week	1212 (23.5)	6.53 (5.55, 7.68)***	6.47 (5.50, 7.61)***	6.42 (5.46, 7.56)***
P		< 0.001	< 0.001	< 0.001
Global sleep quality				
Good (PSQI< 6)	11,432 (2.8)	1.00	1.00	1.00
Poor (PSQI≥6)	5356 (10.7)	3.60 (3.12, 4.17)***	3.56 (3.08, 4.12)***	3.56 (3,07, 4.11)***
P		< 0.001	< 0.001	< 0.001

Model 1: adjusting for sex, age, education level, occupation, personal income, smoking status, alcohol consumption, physical activity, support from family members, and contacts with relatives and friends;

Model 2: additionally adjusting for BMI, presence of chronic diseases, and self-rated health;

Model 3: additionally adjusting for daytime napping or nighttime sleep quality

* *P* < 0.05, *** *P* < 0.001

Association of daytime napping with depressive symptoms varied by sleep quality.

As significant interaction was found (*P* = 0.04), analyses of napping with depressive symptoms were further stratified by sleep quality. Table 3 shows that, in total sample, compared to non-nappers, after adjustment for potential confounders (Model 2), nappers had higher odds of depressive symptoms, with OR (95% CI) of 1.28 (1.11–1.49). Further adjustment for nighttime sleep quality in Model 3, the association remained the same. As significant interaction between daytime napping and sleep quality was found (P for interaction = 0.042), stratification analysis by sleep quality was performed. In participants with good sleep quality, compared to non-nappers, nappers had significantly higher odds of depressive symptoms, with OR (95% CI) being 1.57 (1.23–2.01). No association between napping and depressive symptoms was found in participants with poor sleep quality (OR (95% CI): 1.13 (0.94–1.36)).

**Table 3:** Prevalence and adjusted OR (95% CI) of depressive symptoms by daytime napping

Variables	Depressive symptoms (%)	Model 1	Model 2	Model 3
Total sample				
Daytime napping				
Non-nappers	6699 (4.6)	1.00	1.00	1.00
Nappers	10,087 (5.7)	1.31 (1.13, 1.51)***	1.28 (1.11, 1.49)**	1.28 (1.10, 1.48)**
Good-quality sleepers				
Daytime napping				–
Non-nappers	4595 (2.1%)	1.00	1.00	–
Nappers	6835 (3.2%)	1.60 (1.25, 2.04)***	1.57 (1.23, 2.01)***	–
Poor-quality sleepers				
Daytime napping				–
Non-nappers	2104 (10.1%)	1.00	1.00	–
Nappers	3252 (11.0%)	1.15 (0.96, 1.39)	1.13 (0.94, 1.36)	–

Model 1: adjusting for sex, age, education level, occupation, personal income, smoking status, alcohol consumption, physical activity, support from family members, and contacts with relatives and friends;

Model 2: additionally adjusting for BMI, presence of chronic diseases, and self-rated health;

Model 3: additionally adjusting for daytime napping or nighttime sleep quality

* *P* < 0.05, **P < 0.01, *** *P* < 0.001

### Supplementary analysis

Results of hierarchical regression models remained the same to the main analysis (Supplementary Table 1 and Supplementary Table 2). After excluding 584 participants with sleep apnea from the analysis, the results remained the same (Supplementary Table 3 and Supplementary Table 4). Sex stratification analysis was also conducted to explore any variation by sex. We found that in men and women, the associations of longer napping duration, daily napping, and poorer nighttime sleep quality and its components with depressive symptoms remained (Supplementary Table 5 and Supplementary Table 6). In addition, nighttime sleep quality stratification analysis was conducted to explore whether the associations of napping duration and frequency with depressive symptoms varied by nighttime sleep quality. In good-quality sleepers, those with daily napping and any napping duration had higher odds of depressive symptoms. In poor-quality sleepers, the associations of daily napping and depressive symptoms attenuated and became non-significant after adjustment. For napping duration, significantly higher odds of depressive symptoms were observed in those with napping duration of > 90 min, but not in those with napping duration of < 60 min or 60–90 min (Supplementary Table 7). In addition, poor-quality sleepers had relatively higher prevalence of daytime napping habits, including daily napping and longer napping duration (≥60 min) than good-quality sleepers (Supplementary Table 8). Although we found no evidence that the association of different specific sleep disturbances (including subjective sleep quality, sleep latency, sleep duration, and sleep efficiency) with depressive symptoms varied by daytime napping (P for interactions from 0.12 to 0.52), daytime napping stratification analyses was also conducted to explore any variation by daytime napping. Both in non-nappers and nappers, participants with poorer subjective sleep quality, longer sleep latency, shorter sleep duration, or lower sleep efficiency had higher odds of depressive symptoms. These associations became weaker in nappers than non-nappers. (Supplementary Table 9). Participants with poorer subjective sleep quality had higher odds of depressive symptoms. In participants with napping duration of < 60 min, non-significant associations were observed in those with sleep latency of ≤60 min, shorter sleep duration (≤6 h/day), and lower sleep efficiency, while the associations remained in those with napping duration of 60–90 min and > 90 min, respectively (Supplementary Table 10). Similar results were observed in napping frequency stratification analyses, whatever napping frequency, participants with poorer subjective sleep quality had higher odds of depressive symptoms, and daily nappers with longer sleep latency, shorter sleep duration, or lower sleep efficiency had higher odds of depressive symptoms than those with good subjective sleep quality (Supplementary Table 11).

## Discussion

In this population-based cross-sectional study of 16,786 older Chinese, we found that nappers had significantly higher odds of depressive symptoms than non-nappers, especially in those with extreme napping frequency (daily napping) and long napping duration. Significantly higher odds of depressive symptoms were found in those with poorer nighttime sleep quality. Furthermore, we also showed that the association of napping with depressive symptoms was stronger in good-quality sleepers than that in poor sleepers. As daytime napping may compensate for the detrimental effect of poor nighttime sleep, the association of napping and depressive symptoms in poor-quality sleepers attenuated and became non-significant.

Our most noteworthy finding is the positive association between napping and depressive symptoms was more pronounced in good-quality sleepers than that in poor-quality sleepers. The results indicated that the association of napping with depressive symptoms was modified by sleep quality. Thus, the association of napping with depressive symptoms should be considered and analyzed together with nighttime sleep quality.

Our findings of the association of napping with depressive symptoms were consistent with several previous studies. Two prospective cohort studies of American showed that frequent nappers and those with napping of ≥30 min/day had a higher risk of depressive symptoms and depressive mood, respectively [17, 21]. Another two cross-sectional studies using telephone surveys reported similar results [15, 53]. A cross-sectional study on 0.5 million Chinese aged 39–74 years reported a positive association between daytime napping and depression in both men and women [22]. However, some studies reported conflicting results. A longitudinal study of Chinese reported that longer napping duration (≥60 min/day) was associated with lower odds of depressive symptoms than non-napping [24], whereas a Spanish cohort study found non-significant association between short mid-day nap (≤30 min/day) and incident depression during a follow-up of 10 years [23]. Hence, the existing evidence concerning the association of napping with depressive symptoms is conflicting. In addition, our findings of strong association of poor global sleep quality with depressive symptoms were consistent with previous studies in China. Three cross-sectional studies of Chinese consistently showed that, compared to good-quality sleepers, the odds ratio of depressive symptoms was higher in poor-quality sleepers [12, 39, 40], as well as the other seven PSQI components [40]. Additionally, our results were consistent with population-based cross-sectional studies in Korea [10], Singapore [13] and Japan [54], and evidence from meta-analyses on 9 cross-sectional studies and population-based surveys in Europe and America [9, 11, 55].

Daytime napping is very prevalent worldwide [15, 16]. However, the underlying mechanisms that can provide explanations for the associations of daytime napping and sleep quality with depressive symptoms remain unclear. Some considered napping is beneficial. Daytime napping is traditionally regarded as part of healthy lifestyle in China [16, 22], and is believed to relieve stress in Latin America and Mediterranean countries [56]. Appropriate napping may be beneficial as a supplement to nighttime sleep to alleviate fatigue, pain and possible inflammatory processes caused by chronic diseases [15, 57]. However, other studies reported inconsistent results. Several population studies suggested a complicated and bidirectional association of daytime napping [24, 56] and sleep quality [10, 12] with depressive symptoms, which can reinforce each other and contribute to the formation of vicious cycles [39, 56]. It is plausible that daytime napping is a symptom or a predictor for depression, rather than the cause. In addition, disturbing circadian rhythms is one of the most harmful effects of daytime napping [58]. The amount of sleep needed by each person is usually constant. Changes in sleep patterns, especially sleep architecture and depth, are considered part of the normal aging process. The timing of sleep–wake cycles is regulated by two interacting regulatory systems: the sleep–wake homeostatic drive and the internal circadian clock [59]. As people age, the internal clock becomes less efficient [60], including the reduction of the amplitude of circadian oscillation in all the physiological parameters, such as the melatonin level [61, 62], which may result in interrupted sleep, falling asleep earlier, and/or waking up earlier in the morning [63]. Decline in the efficiency of the central master clock (the suprachiasmatic nucleus in the hypothalamus) may be the key element responsible for this age-related decline.

Due to frequent arousals, older people have difficulty falling asleep and staying asleep. Therefore, in states of sleep rhythm imbalance, short nighttime sleep duration could induce a decline in daytime functioning, psychomotor performance, and cognitive functioning, as well as fatigue and reduction of attention [64]. Daytime napping may provide them another sleep opportunity to enhance sleep quality and probably improve mental health [37]. Older people with physical tiredness and psychological fatigue but without daytime napping may increase the risk of circadian disorder and alteration of hormone levels [65]. Furthermore, lying-in bed was considered as a stress coping and compensating mechanism [66], depressed people may tend to spent more time in bed during daytime in order to recover from the negative feelings of fatigue and tiredness [12]. However, in good-quality sleepers, given relatively constant sleep duration needed, more napping may deprive the nighttime sleep. Previous studies showed that, frequent and longer duration of napping were associated with increased sleep fragmentation [67, 68] and poorer sleep quality [68, 69], which might subsequently lead to mental disorders. In addition, extreme napping habits was linked to higher mental stress and lower physical functions [66]. The above reports may explain our finding that in good-quality sleepers, the combination of daytime napping might disturb the circadian rhythms, which might lead to higher odds of depressive symptoms, whereas for poor-quality sleepers, daytime napping may be a supplement to nighttime sleep loss, so that no significant higher odd of depressive symptoms was found.

Strengths of our study included the comprehensive assessment and consideration of napping and sleep quality, as well as multiple potential confounders and effect modifiers (i.e., sleep quality). However, our study had some limitations. First, objective measures of sleep quality such as using polysomnographic (PSG) recordings were not available. Second, GDS-15 is a screening tool, rather than a clinical diagnostic tool for depression. Hence, misclassification might lead to underestimation of the strength of associations. Third, as other large population-based cohorts in the world, not all participants attended the repeated examination, although more than 60% of the participants returned and were included in the current study. Moreover, as reported in our previous study [70], compared with non-respondents, GBCS participants who returned for follow-up were younger, had higher educational attainment or were married compared with those excluded, although the differences, as suggested by the Cohen’s w, were small (all < 0.3). There were no material differences in other lifestyle variables and co-morbidity (all Cohen’s w < 0.1). Therefore, although our current samples were older and could have more underlying illnesses [19, 20], and survivor bias could not be completely ruled out, the impact of non-response should not be a major concern. Finally, as this study was cross-sectional, reverse causality could not be ruled out and the causal inference between napping and depression could not be confirmed. Mendelian randomization studies may be useful in clarifying the causal relations between them.

## Conclusions

Both daytime napping and poor global nighttime sleep quality were significantly associated with higher odds of depressive symptoms. The positive association of napping and depressive symptoms was stronger in good-quality sleepers, which has not been reported previously and deserves further attention and clarification.

### Supplementary Information

**Additional file 1.**

## Data Availability

Ethical approval in place allows us to share data on requests. Please directly send such requests to the Guangzhou Biobank Cohort Study Data Access Committee (gbcsdata@hku.hk).

## Abbreviations

GBCS: Guangzhou Biobank Cohort Study
PSQI: Pittsburgh Sleep Quality Index
GDS-15: The 15-item Geriatric Depression Scale
OR: Odds ratio
CI: confidence intervals
BMI: Body mass index
SD: Standard deviation
UK: United Kingdom
