# Supplementary material for: Associations of daytime napping and nighttime sleep quality with depressive symptoms in older Chinese: the Guangzhou biobank cohort study
Source: BMC Geriatr. 2023 Dec 19;23:875. doi: 10.1186/s12877-023-04579-6 (PMC10731710; doi:10.1186/s12877-023-04579-6)
Supplement: Supplementary file 1 — Additional file 1. [file 12877_2023_4579_MOESM1_ESM.docx]

**Title:** Associations of daytime napping and nighttime sleep quality with depressive symptoms in older Chinese: the Guangzhou Biobank Cohort Study

**Supplementary Table 1**. Prevalence and adjusted OR (95% CI) of depressive symptoms by groups of napping frequency and duration, and Pittsburgh Sleep Quality Index components and global sleep quality

| Variables | Depressive symptoms (%) | Model 1 | Model 2 | Model 3 | Model 4 | Model 5 |  |
| --- | --- | --- | --- | --- | --- | --- | --- |
| Napping frequency |  |  |  |  |  |  |  |
| Non-nappers | 6,699 (4.6) | 1.00 | 1.00 | 1.00 | 1.00 | 1.00 |  |
| 1-3 days/week | 2,306 (4.8) | 1.11 (0.88, 1.39) | 1.12 (0.89, 1.41) | 1.13 (0.90, 1.41) | 1.11 (0.89, 1.40) | 1.11 (0.88, 1.40) |  |
| 4-6 days/week | 1,500 (4.6) | 1.10 (0.84, 1.44) | 1.14 (0.87, 1.49) | 1.14 (0.87, 1.50) | 1.12 (0.85, 1.47) | 1.10 (0.83, 1.45) |  |
| Daily | 6281 (6.4) | **1.38 (1.18, 1.61)***** | **1.42 (1.21, 1.66)***** | **1.41 (1.20, 1.65)***** | **1.38 (1.18, 1.62)***** | **1.38 (1.17, 1.62)***** |  |
| P for trend |  | <0.001 | <0.001 | <0.001 | <0.001 | <0.001 |  |
| Napping duration |  |  |  |  |  |  |  |
| Non-nappers | 6,699 (4.6) | 1.00 |  | 1.00 | 1.00 | 1.00 |  |
| <60 min | 3,311 (5.4) | 1.21 (1.00, 1.46) | **1.25 (1.03, 1.51)*** | **1.24 (1.02, 1.51)*** | **1.23 (1.01, 1.49)*** | **1.22 (1.01, 1.49)*** |  |
| 60-90 min | 5,561 (5.5) | **1.23 (1.04, 1.45)*** | **1.26 (1.07, 1.49)**** | **1.26 (1.08, 1.49)**** | **1.23 (1.04, 1.46)*** | **1.24 (1.03, 1.45)*** |  |
| >90 min | 1,093 (8.0) | **1.73 (1.34, 2.22)***** | **1.71 (1.33, 2.20)***** | **1.72 (1.33, 2.21)***** | **1.67 (1.30, 2.16)***** | **1.68 (1.30, 2.18)***** |  |
| P for trend |  | <0.001 | <0.001 | <0.001 | <0.001 | <0.001 |  |
| Subjective sleep quality | |  |  |  |  |  |  |
| Good | 14,213 (4.1) | 1.00 | 1.00 | 1.00 | 1.00 | 1.00 |  |
| Poor | 2,575(12.2) | **3.17 (2.74, 3.68)***** | **3.11 (2.68, 3.61)***** | **3.02 (2.60, 3.50)***** | **2.97 (2.55, 2.45)***** | **2.98 (2,57, 3.47)***** |  |
| P |  | <0.001 | <0.001 | <0.001 | <0.001 | <0.001 |  |
| Sleep latency |  |  |  |  |  |  |  |
| ≤30 min | 14,544 (4.5) | 1.00 | 1.00 | 1.00 | 1.00 | 1.00 |  |
| 31-60 min | 1,670 (8.6) | **1.81 (1.50, 2.20)***** | **1.80 (1.48, 2.18)***** | **1.74 (1.43, 2.11)***** | **1.71 (1.41, 2.08)***** | **1.70 (1.40, 2.06)***** |  |
| >60 min | 574 (14.6) | **3.17 (2.47, 4.08)***** | **3.14 (2.44, 4.04)***** | **3.03 (2.35, 3.90)***** | **3.02 (2.34, 3.89)***** | **3.01 (2.33, 3.88)***** |  |
| P for trend |  | <0.001 | <0.001 | <0.001 | <0.001 | <0.001 |  |
| Sleep duration |  |  |  |  |  |  |  |
| <5 h/day | 893 (11.0) | **2.35 (1.84, 3.00)***** | **2.40 (1.88, 3.06)***** | **2.32 (1.81, 2.97)***** | **2.28 (1.78, 2.92)***** | **2.31 (1.80, 2.96)***** |  |
| 5-6 h/day | 6,503 (6.7) | **1.59 (1.37, 1.85)***** | **1.59 (1.37, 1.85)***** | **1.58 (1.36, 1.84)***** | **1.59 (1.36, 1.85)***** | **1.59 (1.36, 1.85)***** |  |
| 7-8 h/day | 7,917 (4.0) | 1.00 | 1.00 | 1.00 | 1.00 | 1.00 |  |
| >8 h/day | 843 (2.7) | 0.65 (0.42, 1.00) | 0.67 (0.43, 1.03) | 0.67 (0.43, 1.03) | 0.66 (0.43, 1.03) | 0.67 (0.44, 1.04) |  |
| P for trend |  | <0.001 | <0.001 | <0.001 | <0.001 | <0.001 |  |
| Sleep efficiency |  |  |  |  |  |  |  |
| ≥85% | 11,348 (4.1) | 1.00 | 1.00 | 1.00 | 1.00 | 1.00 |  |
| 75-84% | 2,664 (6.9) | **1.58 (1.32, 1.89)***** | **1.55 (1.29, 1.85)***** | **1.54 (1.29, 1.85)***** | **1.54 (1.29, 1.85)***** | **1.54 (1.28, 1.85)***** |  |
| 65-74% | 1,403 (8.3) | **1.89 (1.53, 2.35)***** | **1.87 (1.51, 2.32)***** | **1.88 (1.51, 2.34)***** | **1.85 (1.49, 2.29)***** | **1.86 (1.50, 2.31)***** |  |
| <65% | 1,373 (9.3) | **2.02 (1.64, 2.49)***** | **1.98 (1.60, 2.44)***** | **1.98 (1.60, 2.45)***** | **1.94 (1.57, 2.40)***** | **1.94 (1.57, 2.39)***** |  |
| P for trend |  | <0.001 | <0.001 | <0.001 | <0.001 | <0.001 |  |
| Sleep disturbances |  |  |  |  |  |  |  |
| Not at all/<once per week | 16,416 (4.7) | 1.00 | 1.00 | 1.00 | 1.00 | 1.00 |  |
| At least once per week | 372 (29.8) | **7.29 (5.72, 9.29)***** | **6.96 (5.45, 8.88)***** | **6.51 (5.08, 8.34)***** | **6.41 (5.00, 8.21)***** | **6.35 (4.95, 8.14)***** |  |
| P |  | <0.001 | <0.001 | <0.001 | <0.001 | <0.001 |  |
| Sleep medications |  |  |  |  |  |  |  |
| Not at all/<once per week | 16,415 (5.1) | 1.00 | 1.00 | 1.00 | 1.00 | 1.00 |  |
| At least once per week | 373 (13.4) | **2.88 (2.10, 3.94)***** | **2.94 (2.14, 4.03)***** | **2.99 (2.18, 4.12)***** | **2.88 (2.09, 3.96)***** | **2.91 (2.12, 4.01)***** |  |
| P |  | <0.001 | <0.001 | <0.001 | <0.001 | <0.001 |  |
| Daytime dysfunction |  |  |  |  |  |  |  |
| Not at all/<once per week | 15,576 (3.9) | 1.00 | 1.00 | 1.00 | 1.00 | 1.00 |  |
| At least once per week | 1,212 (23.5) | **6.86 (5.84, 8.05)***** | **6.70 (5.71, 7.87)***** | **6.53 (5.55, 7.68)***** | **6.47 (5.50, 7.61)***** | **6.42 (5.46, 7.56)***** |  |
| P |  | <0.001 | <0.001 | <0.001 | <0.001 | <0.001 |  |
| Global sleep quality | |  |  |  |  |  |  |
| Good (PSQI<6) | 11,432 (2.8) | 1.00 | 1.00 | 1.00 | 1.00 | 1.00 |  |
| Poor (PSQI≥6) | 5,356 (10.7) | **3.78 (3.27, 4.36)***** | **3.70 (3.20, 4.27)***** | **3.60 (3.12, 4.17)***** | **3.56 (3.08, 4.12)***** | **3.56 (3,07, 4.11)***** |  |
| P |  | <0.001 | <0.001 | <0.001 | <0.001 | <0.001 |  |

Model 1: adjusting for sex, age, education level, occupation, personal income;

Model 2: additionally adjusting for smoking status, alcohol consumption, physical activity;

Model 3: additionally adjusting for support from family members, and contacts with relatives and friends;

Model 4: additionally adjusting for BMI, presence of chronic diseases, and self-rated health;

Model 5: additionally adjusting for daytime napping or nighttime sleep quality.

* P<0.05, ** P <0.01, *** P <0.001.

**Supplementary Table 2**. Prevalence and adjusted OR (95% CI) of depressive symptoms by daytime napping

| Variables | Depressive symptoms (%) | Model 1 | Model 2 | Model 3 | Model 4 | Model 5 |
| --- | --- | --- | --- | --- | --- | --- |
|  |  | Total sample | | | | |
| Daytime napping |  |  |  |  |  |  |
| Non-nappers | 6,699 (4.6) | 1.00 | 1.00 | 1.00 | 1.00 | 1.00 |
| Nappers | 10,087 (5.7) | **1.28 (1.10, 1.47)**** | **1.31 (1.13, 1.51)***** | **1.31 (1.13, 1.51)***** | **1.28 (1.11, 1.49)**** | **1.28 (1.10, 1.48)**** |
|  |  | Good-quality sleepers | | | | |
| Daytime napping |  |  |  |  |  | - |
| Non-nappers | 4,595 (2.1%) | 1.00 | 1.00 | 1.00 | 1.00 | - |
| Nappers | 6,835 (3.2%) | **1.54 (1.21, 1.97)***** | **1.60 (1.25, 2.05)***** | **1.60 (1.25, 2.04)***** | **1.57 (1.23, 2.01)***** | - |
|  |  | Poor-quality sleepers | | | | |
| Daytime napping |  |  |  |  |  | - |
| Non-nappers | 2,104 (10.1%) | 1.00 | 1.00 | 1.00 | 1.00 | - |
| Nappers | 3,252 (11.0%) | 1.13 (0.94, 1.35) | 1.15 (0.96, 1.39) | 1.15 (0.96, 1.39) | 1.13 (0.94, 1.36) | - |

Model 1: adjusting for sex, age, education level, occupation, personal income;

Model 2: additionally adjusting for smoking status, alcohol consumption, physical activity;

Model 3: additionally adjusting for support from family members, and contacts with relatives and friends;

Model 4: additionally adjusting for BMI, presence of chronic diseases, and self-rated health;

Model 5: additionally adjusting for daytime napping or nighttime sleep quality.

* P<0.05, ** P <0.01, *** P <0.001.

**Supplementary Table 3**. Prevalence and adjusted OR (95% CI) of depressive symptoms by groups of napping frequency and duration, and Pittsburgh Sleep Quality Index components and global sleep quality, after excluding those with sleep apnea

| Variables | Depressive symptoms (%) | Model 1 | Model 2 | Model 3 |
| --- | --- | --- | --- | --- |
| Napping frequency |  |  |  |  |
| Non-nappers | 6,496 (4.4) | 1.00 | 1.00 | 1.00 |
| 1-3 days/week | 2,223 (4.6) | 1.16 (0.92, 1.47) | 1.15 (0.91, 1.45) | 1.15 (0.90, 1.45) |
| 4-6 days/week | 1,438 (4.2) | 1.09 (0.82, 1.46) | 1.07 (0.80, 1.43) | 1.05 (0.79, 1.41) |
| daily | 6,045 (6.1) | **1.41 (1.20, 1.66)***** | **1.39 (1.18, 1.64)***** | **1.38 (1.17, 1.63)***** |
| P for trend |  | <0.001 | <0.001 | <0.001 |
| Napping duration |  |  |  |  |
| Non-nappers | 6,496 (4.4) | 1.00 | 1.00 | 1.00 |
| <60 min | 3,225 (5.0) | 1.21 (0.99, 1.48) | 1.20 (0.98, 1.47) | 1.19 (0.97, 1.46) |
| 60-90 min | 5,425 (5.3) | **1.26 (1.06, 1.50)**** | **1.24 (1.04, 1.47)*** | **1.23 (1.03, 1.47)*** |
| >90 min | 1,056 (8.0) | **1.82 (1.41, 2.36)***** | **1.77 (1.37, 2.30)***** | **1.79 (1.37, 2.33)***** |
| P for trend |  | <0.001 | <0.001 | <0.001 |
| Subjective sleep quality | |  |  |  |
| Good | 13,787 (3.9) | 1.00 | 1.00 | 1.00 |
| Poor | 2,415 (11.7) | **3.05 (2.61, 3.57)***** | **3.01 (2.57, 3.53)***** | **3.03 (2.59, 3.55)***** |
| P |  | <0.001 | <0.001 | <0.001 |
| Sleep latency |  |  |  |  |
| ≤30 min | 14,079 (4.3) | 1.00 | 1.00 | 1.00 |
| 31-60 min | 1,590 (8,3) | **1.74 (1.42, 2.13)***** | **1.72 (1.40, 2.10)***** | **1.71 (1.39, 2.09)***** |
| >60 min | 533 (13.9) | **2.97 (2.27, 3.88)***** | **2.96 (2.26, 3.87)***** | **2.97 (2.27, 3.88)***** |
| P for trend |  | <0.001 | <0.001 | <0.001 |
| Sleep duration |  |  |  |  |
| <5 h/day | 845 (10.5) | **2.28 (1.76, 2.95)***** | **2.24 (1.73, 2.90)***** | **2.27 (1.75, 2.94)***** |
| 5-6 h/day | 6,224 (6.4) | **1.54 (1.32, 1.81)***** | **1.54 (1.32, 1.81)***** | **1.54 (1.32, 1.81)***** |
| 7-8 h/day | 7,701 (3.8) | 1.00 | 1.00 | 1.00 |
| >8 h/day | 827 (2.2) | **0.55 (0.34, 0.90)*** | **0.55 (0.34, 0.89)*** | **0.56 (0.34, 0.90)*** |
| P for trend |  | 0.001 | 0.001 | 0.001 |
| Sleep efficiency |  |  |  |  |
| ≥85% | 11,007 (3.8) | 1.00 | 1.00 | 1.00 |
| 75-84% | 2,555 (6.6) | **1.55 (1.28, 1.87)***** | **1.55 (1.28, 1.87)***** | **1.54 (1.28, 1.86)***** |
| 65-74% | 1,345 (8.0) | **1.87 (1.50, 2.35)***** | **1.84 (1.47, 2.31)***** | **1.85 (1.48, 2.33)***** |
| <65% | 1,295 (9.2) | **2.04 (1.64, 2.54)***** | **2.00 (1.61, 2.50)***** | **2.00 (1.61, 2.49)***** |
| P for trend |  | <0.001 | <0.001 | <0.001 |
| Sleep disturbances |  |  |  |  |
| Not at all/<once per week | 15,883 (4.6) | 1.00 | 1.00 | 1.00 |
| At least once per week | 319 (29.5) | **6.91 (5.29, 9.01)***** | **6.80 (5.21, 8.88)***** | **6.72 (5.15, 8.78)***** |
| P |  | <0.001 | <0.001 | <0.001 |
| Sleep medications |  |  |  |  |
| Not at all/<once per week | 15,848 (4.9) | 1.00 | 1.00 | 1.00 |
| At least once per week | 354 (12.7) | **2.96 (2.12, 4.13)***** | **2.86 (2.04, 4.00)***** | **2.91 (2.08, 4.06)***** |
| P |  | <0.001 | <0.001 | <0.001 |
| Daytime dysfunction |  |  |  |  |
| Not at all/<once per week | 15,083 (3.8) | 1.00 | 1.00 | 1.00 |
| At least once per week | 1,119 (22.3) | **6.29 (5.30, 7.45)***** | **6.23 (5.26, 7.39)***** | **6.20 (5.22, 7.35)***** |
| P |  | <0.001 | <0.001 | <0.001 |
| Global sleep quality | |  |  |  |
| Good (PSQI<6) | 11,149 (2.7) | 1.00 | 1.00 | 1.00 |
| Poor (PSQI≥6) | 5,053 (10.3) | **3.58 (3.08, 4.16)***** | **3.54 (3.05, 4.12)***** | **3.54 (3.04, 4.12)***** |
| P |  | <0.001 | <0.001 | <0.001 |

Model 1: adjusting for sex, age, education level, occupation, personal income, smoking status, alcohol consumption, physical activity, support from family members, and contacts with relatives and friends;

Model 2: additionally adjusting for BMI, presence of chronic diseases, and self-rated health;

Model 3: additionally adjusting for daytime napping or nighttime sleep quality.

* P<0.05, *** P <0.001.

**Supplementary Table 4**. Prevalence and adjusted OR (95% CI) of depressive symptoms by daytime napping, after excluding those with sleep apnea

| Variables | Depressive symptoms (%) | Model 1 | Model 2 | Model 3 |
| --- | --- | --- | --- | --- |
| Total sample | | | | |
| Daytime napping |  |  |  |  |
| Non-nappers | 6,496 (4.4) | 1.00 | 1.00 | 1.00 |
| Nappers | 9,706 (5.5) | **1.31 (1.13, 1.52)**** | **1.29 (1.11, 1.50)**** | **1.28 (1.10, 1.50)**** |
| Good-quality sleepers | | | | |
| Daytime napping |  |  |  | - |
| Non-nappers | 4,499 (2.1) | 1.00 | 1.00 | - |
| Nappers | 6,650 (3.1) | **1.56 (1.21, 2.01)**** | **1.54 (1.19, 1.98)**** | - |
| Poor-quality sleepers | | | | |
| Daytime napping |  |  |  | - |
| Non-nappers | 1,997 (9.6) | 1.00 | 1.00 | - |
| Nappers | 3,056 (10.7) | 1.17 (0.98, 1.42) | 1.15 (0.94, 1.39) | - |

Model 1: adjusting for sex, age, education level, occupation, personal income, smoking status, alcohol consumption, physical activity, support from family members, and contacts with relatives and friends;

Model 2: additionally adjusting for BMI, presence of chronic diseases, and self-rated health;

Model 3: additionally adjusting for daytime napping or nighttime sleep quality.

* P<0.05, **P<0.01, *** P <0.001.

**Supplementary Table 5**. Sex-specific prevalence and adjusted OR (95% CI) of depressive symptoms by groups of napping frequency and duration, Pittsburgh Sleep Quality Index components and global sleep quality

| Gender | Variables | Depressive symptoms (%) | Model 1 | Model 2 | Model 3 |
| --- | --- | --- | --- | --- | --- |
| Men (N=4,541) | Napping frequency |  |  |  |  |
|  | Non-nappers | 1,344 (3.7) | 1.00 | 1.00 | 1.00 |
|  | 1-3 days/week | 523 (3.6) | 1.09 (0.63, 1.88) | 1.07 (0.61, 1.85) | 1.04 (0.59, 1.83) |
|  | 4-6 days/week | 451 (4.2) | 1.27 (0.73, 2.20) | 1.24 (0.71, 2.15) | 1.19 (0.68, 2.08) |
|  | daily | 2,223 (6.3) | **1.81 (1.29, 2.55)**** | **1.78 (1.26, 2.50)**** | **1.79 (1.27, 2.54)**** |
|  | P for trend |  | <0.001 | <0.001 | <0.001 |
|  | Napping duration |  |  |  |  |
|  | Non-nappers | 1,344 (3.7) | 1.00 | 1.00 | 1.00 |
|  | <60 min | 829 (5.2) | **1.55 (1.01, 2.38)*** | 1.51 (0.98, 2.32) | 1.50 (0.97, 2.33) |
|  | 60-90 min | 1,981 (5.5) | **1.57 (1.10, 2.23)*** | **1.54 (1.08, 2.19)*** | **1.54 (1.07, 2.20)*** |
|  | >90 min | 387 (7.2) | **1.95 (1.20, 3.18)**** | **1.91 (1.17, 3.12)*** | **1.90 (1.15, 3.14)*** |
|  | P for trend |  | 0.003 | 0.004 | 0.006 |
|  | Subjective sleep quality | |  |  |  |
|  | Good | 4,027 (4.1) | 1.00 | 1.00 | 1.00 |
|  | Poor | 514 (12.5) | **3.03 (2.21, 4.17)***** | **2.95 (2.14, 4.06)***** | **3.03 (2.20, 4.17)***** |
|  | P |  | <0.001 | <0.001 | <0.001 |
|  | Sleep latency |  |  |  |  |
|  | ≤30 min | 4,067 (4.3) | 1.00 | 1.00 | 1.00 |
|  | 31-60 min | 361 (9.4) | **2.02 (1.35, 3.02)**** | **1.99 (1.33, 2.97)**** | **2.00 (1.34, 2.99)**** |
|  | >60 min | 113 (16.8) | **4.07 (2.38, 6.98)***** | **3.90 (2.27, 6.71)***** | **4.20 (2.44, 7.24)***** |
|  | P for trend |  | <0.001 | <0.001 | <0.001 |
|  | Sleep duration |  |  |  |  |
|  | <5 h/day | 206 (10.2) | **2.63 (1.56, 4.44)***** | **2.58 (1.53, 4.37)***** | **2.58 (1.52, 4.38)***** |
|  | 5-6 h/day | 1,763 (6.8) | **1.92 (1.43, 2.61)***** | **1.94 (1.44, 2.63)***** | **1.93 (1.43, 2.62)***** |
|  | 7-8 h/day | 2,157 (3.6) | 1.00 | 1.00 | 1.00 |
|  | >8 h/day | 231 (3.0) | 0.88 (0.40, 1.96) | 0.89 (0.40, 1.97) | 0.94 (0.42, 2.10) |
|  | P for trend |  | 0.002 | 0.002 | 0.001 |
|  | Sleep efficiency |  |  |  |  |
|  | ≥85% | 3,137 (3.7) | 1.00 | 1.00 | 1.00 |
|  | 75-84% | 709 (6.9) | **1.93 (1.35, 2.74)***** | **1.93 (1.5, 2.75)***** | **1.94 (1.36, 2.77)***** |
|  | 65-74% | 371 (8.9) | **2.35 (1.54, 3.58)***** | **2.30 (1.51, 2.51)***** | **2.34 (1.53, 3.57)***** |
|  | <65% | 324 (9.6) | **2.56 (1.67, 3.94)***** | **2.48 (1.61, 3.81)***** | **2.44 (1.58, 3.77)***** |
|  | P for trend |  | <0.001 | <0.001 | <0.001 |
|  | Sleep disturbances |  |  |  |  |
|  | Not at all/<once per week | 4,455 (4.4) | 1.00 | 1.00 | 1.00 |
|  | At least once per week | 86 (39.5) | **11.02 (6.74, 18.02)***** | **10.75 (6.56, 17.61)***** | **10.67 (6.49, 17.53)***** |
|  | P |  | <0.001 | <0.001 | <0.001 |
|  | Sleep medications |  |  |  |  |
|  | Not at all/<once per week | 4,451 (4.9) | 1.00 | 1.00 | 1.00 |
|  | At least once per week | 90 (11.1) | **2.62 (1.31, 5.25)**** | **2.32 (1.15, 4.68)*** | **2.47 (1.22, 4.99)*** |
|  | P |  | 0.006 | 0.019 | 0.012 |
|  | Daytime dysfunction |  |  |  |  |
|  | Not at all/<once per week | 4,267 (3.8) | 1.00 | 1.00 | 1.00 |
|  | At least once per week | 274 (25.2) | **7.59 (5.44, 10.59)***** | **7.51 (5.37, 10.50)***** | **7.41 (5.29, 10.38)***** |
|  | P |  | <0.001 | <0.001 | <0.001 |
|  | Global sleep quality | |  |  |  |
|  | Good (PSQI<6) | 3,305 (2.7) | 1.00 | 1.00 | 1.00 |
|  | Poor (PSQI≥6) | 1,236 (11.3) | **4.07 (3.07, 5.41)***** | **4.00 (3.01, 5.31)***** | **4.03 (3.03, 5.36)***** |
|  | P |  | <0.001 | <0.001 | <0.001 |
| Women (N=12,245) | Napping frequency |  |  |  |  |
|  | Non-nappers | 5,355 (4.9) | 1.00 | 1.00 | 1.00 |
|  | 1-3 days/week | 1,783 (5.1) | 1.13 (0.88, 1.46) | 1.12 (0.88, 1.44) | 1.13 (0.88, 1.45) |
|  | 4-6 days/week | 1,049 (4.8) | 1.13 (0.82, 1.54) | 1.09 (0.80, 1.50) | 1.09 (0.79, 1.50) |
|  | daily | 4,058 (6.4) | **1.29 (1.07, 1.54)**** | **1.26 (1.05, 1.52)*** | **1.25 (1.04, 1.51)*** |
|  | P for trend |  | 0.005 | 0.009 | 0.016 |
|  | Napping duration |  |  |  |  |
|  | Non-nappers | 5,355 (4.9) | 1.00 | 1.00 | 1.00 |
|  | <60 min | 2,528 (5.4) | 1.17 (0.94, 1.45) | 1.16 (0.93, 1.44) | 1.16 (0.93, 1.44) |
|  | 60-90 min | 3,647 (5.6) | 1.17 (0.96, 1.41) | 1.14 (0.94, 1.39) | 1.13 (0.93, 1.38) |
|  | >90 min | 715 (8.4) | **1.70 (1.26, 2.29)**)** | **1.65 (1.22, 2.23)**** | **1.67 (1.23, 2.26)**** |
|  | P for trend |  | 0.002 | 0.004 | 0.006 |
|  | Subjective sleep quality |  |  |  |  |
|  | Good | 10,184 (4.0) | 1.00 | 1.00 | 1.00 |
|  | Poor | 2,061 (12.1) | **3.04 (2.57, 3.61)***** | **3.01 (2.54, 3.58)***** | **3.02 (2.54, 3.59)***** |
|  | P |  | <0.001 | <0.001 | <0.001 |
|  | Sleep latency |  |  |  |  |
|  | ≤30 min | 10,475 (4.6) | 1.00 | 1.00 | 1.00 |
|  | 31-60 min | 1,309 (8.4) | **1.64 (1.32, 2.05)***** | **1.62 (1.30, 2.03)***** | **1.61 (1.29, 2.02)***** |
|  | >60 min | 461 (14.1) | **2.82 (2.11, 3.76)***** | **2.81 (2.11, 3.75)***** | **2.80 (2.09, 3.73)***** |
|  | P for trend |  | <0.001 | <0.001 | <0.001 |
|  | Sleep duration |  |  |  |  |
|  | <5 h/day | 687 (11.2) | **2.26 (1.71, 3.00)***** | **2.23 (1.68, 2.95)***** | **2.26 (1.70, 2.99)***** |
|  | 5-6 h/day | 4,739 (6.7) | **1.49 (1.25, 1.78)***** | **1.49 (1.25, 1.77)***** | **1.49 (1.25, 1.78)***** |
|  | 7-8 h/day | 5,759 (4.1) | 1.00 | 1.00 | 1.00 |
|  | >8 h/day | 612 (2.6) | 0.61 (0.36, 1.02) | 0.60 (0.35, 1.00) | 0.60 (0.36, 1.01) |
|  | P for trend |  | 0.004 | 0.004 | 0.005 |
|  | Sleep efficiency |  |  |  |  |
|  | ≥85% | 8,209 (4.2) | 1.00 | 1.00 | 1.00 |
|  | 75-84% | 1,955 (6.9) | **1.44 (1.17, 1.78)**** | **1.44 (1.17, 1.78)**** | **1.44 (1.17, 1.78)**** |
|  | 65-74% | 1,032 (8.1) | **1.73 (1.34, 2.23)***** | **1.69 (1.31, 2.19)***** | **1.70 (1.32, 2.20)***** |
|  | <65% | 1,049 (9.3) | **1.85 (1.45, 2.35)***** | **1.82 (1.42, 2.32)***** | **1.82 (1.43, 2.32)***** |
|  | P for trend |  | <0.001 | <0.001 | <0.001 |
|  | Sleep disturbances |  |  |  |  |
|  | Not at all/<once per week | 11,959 (4.9) | 1.00 | 1.00 | 1.00 |
|  | At least once per week | 286 (26.6) | **5.43 (4.06, 7.26)***** | **5.36 (4.00, 7.17)***** | **5.33 (3.98, 7.13)***** |
|  | P |  | <0.001 | <0.001 | <0.001 |
|  | Sleep medications |  |  |  |  |
|  | Not at all/<once per week | 11,962 (5.2) | 1.00 | 1.00 | 1.00 |
|  | At least once per week | 283 (14.1) | **3.16 (2.21, 4.53)***** | **3.11 (2.17, 4.45)***** | **3.13 (2.18, 4.48)***** |
|  | P |  | <0.001 | <0.001 | <0.001 |
|  | Daytime dysfunction |  |  |  |  |
|  | Not at all/<once per week | 11,308 (3.9) | 1.00 | 1.00 | 1.00 |
|  | At least once per week | 937 (23.1) | **6.33 (5.25, 7.62)***** | **6.26 (5.19, 7.54)***** | **6.24 (5.17, 7.52)***** |
|  | P |  | <0.001 | <0.001 | <0.001 |
|  | Global sleep quality |  |  |  |  |
|  | Good (PSQI<6) | 8,125 (2.8) | 1.00 | 1.00 | 1.00 |
|  | Poor (PSQI≥6) | 4,120 (10.5) | **3.48 (2.93, 4.12)***** | **3.44 (2.90, 4.08)***** | **3.43 (2.90, 4.07)***** |
|  | P |  | <0.001 | <0.001 | <0.001 |

Model 1: adjusting for age, education level, occupation, personal income, smoking status, alcohol consumption, physical activity, support from family members, and contacts with relatives and friends;

Model 2: additionally adjusting for BMI, presence of chronic diseases, and self-rated health;

Model 3: additionally adjusting for daytime napping or nighttime sleep quality.

* P<0.05, **P<0.01, *** P <0.001.

**Supplementary Table 6.** Prevalence and adjusted OR (95% CI) of depressive symptoms by daytime napping, stratifying by sex

| Gender | Variables | Depressive symptoms (%) | Model 1 | Model 2 | Model 3 |
| --- | --- | --- | --- | --- | --- |
| Men (N=4,543) | Total sample | | | | |
|  | Daytime napping |  |  |  |  |
|  | Non-nappers | 1,344 (3.7) | 1.00 | 1.00 | 1.00 |
|  | Nappers | 3,197 (5.6) | **1.61 (1.16, 2.24)**** | **1.58 (1.14, 2.20)**** | **1.58 (1.13, 2.21)**** |
|  | Good-quality sleepers | | | | |
|  | Daytime napping |  |  |  | - |
|  | Non-nappers | 992 (1.8) | 1.00 | 1.00 | - |
|  | Nappers | 2,313 (3.1) | **1.81 (1.06, 3.09)*** | **1.77 (1.04, 3.03)*** | - |
|  | Poor-quality sleepers | | | | |
|  | Daytime napping |  |  |  | - |
|  | Non-nappers | 352 (9.1) | 1.00 | 1.00 | - |
|  | Nappers | 884 (12.1) | 1.37 (0.89, 2.13) | 1.37 (0.88, 2.13) | - |
| Women (N=12,245) | Total sample | | | | |
|  | Daytime napping |  |  |  |  |
|  | Non-nappers | 5,355 (4.9) | 1.00 | 1.00 | 1.00 |
|  | Nappers | 6,890 (5.8) | **1.23 (1.04, 1.44)*** | **1.20 (1.02, 1.42)*** | **1.20 (1.02, 1.42)*** |
|  | Good-quality sleepers | | | | |
|  | Daytime napping |  |  |  | - |
|  | Non-nappers | 3,603 (2.2) | 1.00 | 1.00 | - |
|  | Nappers | 4,522 (3.3) | **1.52 (1.15, 2.01)**** | **1.50 (1.13, 1.99)**** | - |
|  | Poor-quality sleepers | | | | |
|  | Daytime napping |  |  |  | - |
|  | Non-nappers | 1,752 (10.3) | 1.00 | 1.00 | - |
|  | Nappers | 2,368 (10.6) | 1.08 (0.88, 1.33) | 1.06 (0.86, 1.31) | - |

Model 1: adjusting for age, education level, occupation, personal income, smoking status, alcohol consumption, physical activity, support from family members, and contacts with relatives and friends;

Model 2: additionally adjusting for BMI, presence of chronic diseases, and self-rated health;

Model 3: additionally adjusting for daytime napping or nighttime sleep quality.

* P<0.05, **P<0.01.

**Supplementary Table 7**. Prevalence and adjusted OR (95% CI) of depressive symptoms by groups of napping frequency and duration, stratifying by nighttime sleep quality

| Variables | Depressive symptoms (%) | Model 1 | Model 2 |
| --- | --- | --- | --- |
| Good-quality sleepers | | | |
| Napping frequency |  |  |  |
| Non-nappers | 4,595 (2.1) | 1.00 | 1.00 |
| 1-3 days/week | 1,586 (2.8) | 1.43 (0.99, 2.06) | 1.42 (0.99, 2.04) |
| 4-6 days/week | 1,034 (2.2) | 1.21 (0.76, 1.93) | 1.19 (0.74, 1.89) |
| daily | 4,215 (3.6) | **1.75 (1.34, 2.28)***** | **1.72 (1.32, 2.24)***** |
| P for trend |  | <0.001 | <0.001 |
| Napping duration |  |  |  |
| Non-nappers | 4,595 (2.1) | 1.00 | 1.00 |
| <60 min | 2,269 (3.3) | **1.65 (1.20, 2.25)**** | **1.63 (1.19, 2.23)**** |
| 60-90 min | 3,828 (3.0) | **1.52 (1.15, 2.00)**** | **1.49 (1.13, 1.97)**** |
| >90 min | 738 (4.1) | **1.84 (1.20, 2.81)**** | **1.79 (1.17, 2.74)**** |
| P for trend |  | 0.001 | 0.001 |
| Poor-quality sleepers | | | |
| Napping frequency |  |  |  |
| Non-nappers | 2,104 (10.1) | 1.00 | 1.00 |
| 1-3 days/week | 720 (9.0) | 0.97 (0.72, 1.31) | 0.95 (0.71, 1.28) |
| 4-6 days/week | 466 (9.9) | 1.08 (0.76, 1.52) | 1.06 (0.75, 1.50) |
| daily | 2,066 (12.0) | **1.23 (1.01, 1.51)*** | 1.21 (0.99, 1.49) |
| P for trend |  | 0.040 | 0.058 |
| Napping duration |  |  |  |
| Non-nappers | 2,104 (10.1) | 1.00 | 1.00 |
| <60 min | 1,088 (9.7) | 1.03 (0.80, 1.33) | 1.02 (0.79, 1.31) |
| 60-90 min | 1,800 (10.8) | 1.12 (0.90, 1.39) | 1.10 (0.89, 1.36)) |
| >90 min | 364 (15.9) | **1.67 (1.20, 2.31)**** | **1.62 (1.17, 2.25)**** |
| P for trend |  | 0.014 | 0.024 |

Model 1: adjusting for sex, age, education level, occupation, personal income, smoking status, alcohol consumption, physical activity, support from family members, and contacts with relatives and friends;

Model 2: additionally adjusting for BMI, presence of chronic diseases, and self-rated health;

* P<0.05, **P<0.01, *** P <0.001.

**Supplementary Table 8**. Comparison of napping frequency and duration in good-quality sleepers and poor-quality sleepers

| Variables | Nighttime sleep quality | |
| --- | --- | --- |
|  | Good-quality sleepers | Poor-quality sleepers |
| Daytime napping |  |  |
| Non-nappers | 4,595 (40.2) | 2,104 (39.3) |
| Nappers | 6,835 (59.8) | 3,252 (60.7) |
| Napping frequency |  |  |
| Non-nappers | 4,595 (40.2) | 2,104 (39.3) |
| 1-3 days/week | 1,586 (13.9) | 720 (13.4) |
| 4-6 days/week | 1,034 (9.0) | 466 (8.7) |
| daily | 4,215 (36.9) | 2,066 (38.6) |
| Napping duration |  |  |
| Non-nappers | 4,595 (40.2) | 2,104 (39.3) |
| <60 min | 2,269 (19.9) | 1,088 (20.3) |
| 60-90 min | 3,828 (33.5) | 1,800 (33.6) |
| >90 min | 738 (6.5) | 364 (6.8) |
| Depressive symptoms |  |  |
| GDS-15≤5 | 11,112 (97.2) | 4,785 (89.3) |
| GDS-15>5 | 318 (2.8) | 571 (10.7) |

**Supplementary Table 9**. Prevalence and adjusted OR (95% CI) of depressive symptoms by groups of different specific sleep disturbances, stratifying by daytime napping

| Variables | Depressive symptoms (%) | Model 1 | Model 2 | Depressive symptoms (%) | Model 1 | Model 2 |
| --- | --- | --- | --- | --- | --- | --- |
| **Non-nappers** | | | | **Nappers** | | |
| Subjective sleep quality |  |  |  |  |  |  |
| Good | 5,606 (3.3) | 1.00 | 1.00 | 8,605 (4.5) | 1.00 | 1.00 |
| Poor | 1,093 (11.4) | **3.42 (2.67, 4.38)***** | **3.37 (2.62, 4.32)***** | 1,482 (12.7) | **2.84 (2.35, 3.44)***** | **2.78 (2.30, 3.37)***** |
| P |  | <0.001 | <0.001 |  | <0.001 | <0.001 |
| Sleep latency |  |  |  |  |  |  |
| ≤30 min | 5,826 (3.8) | 1.00 | 1.00 | 8,716 (5.0) | 1.00 | 1.00 |
| 31-60 min | 640 (7.8) | **1.82 (1.31, 2.53)***** | **1.78 (1.28, 2.47)***** | 1,030 (9.1) | **1.65 (1.30, 2.10)***** | **1.62 (1.28, 2.07)***** |
| >60 min | 233 (15.5) | **3.39 (2.27, 5.07)***** | **3.37 (2.25, 5.04)***** | 341 (14.1) | **2.81 (2.02, 3.92)***** | **2.78 (2.00, 3.88)***** |
| P for trend |  | <0.001 | <0.001 |  | <0.001 | <0.001 |
| Sleep duration |  |  |  |  |  |  |
| <5 h/day | 393 (12.2) | **3.13 (2.15, 4.57)***** | **3.16 (2.17, 4.61)***** | 500 (10.0) | **1.89 (1.35, 2.64)***** | **1.83 (1.31, 2.56)***** |
| 5-6 h/day | 2,525 (5.7) | **1.59 (1.22, 2.07)**** | **1.58 (1.21, 2.06)**** | 3,977 (7.4) | **1.58 (1.31, 1.90)***** | **1.58 (1.31, 1.91)***** |
| 7-8 h/day | 3,161 (3.3) | 1.00 | 1.00 | 4,755 (4.4) | 1.00 | 1.00 |
| >8 h/day | 379 (1,9) | 0.55 (0.25, 1.21) | 0.54 (0.25, 1.19) | 464 (3.5) | 0.76 (0.45, 1.28) | 0.75 (0.44, 1.26) |
| P for trend |  | 0.026 | 0.030 |  | 0.001 | 0.001 |
| Sleep efficiency |  |  |  |  |  |  |
| ≥85% | 4,551 (3.4) | 1.00 | 1.00 | 6,795 (4.5) | 1.00 | 1.00 |
| 75-84% | 1,028 (5,7) | **1.49 (1.09, 2.05)*** | **1.47 (1.07, 2.02)***** | 1,636 (7.6) | **1.57 (1.26, 1.95)***** | **1.58 (1.27, 1.98)***** |
| 65-74% | 564 (8.7) | **2.33 (1.65, 3.30)***** | **2.30 (1.63, 3.26)***** | 839 (8.1) | **1.67 (1.26, 2.20)***** | **1.64 (1.24, 2.17)***** |
| <65% | 556 (8.6) | **2.25 (1.58, 3.19)***** | **2.23 (1.57, 3.16)***** | 817 (9.8) | **1.86 (1.42, 2.42)***** | **1.80 (1.38, 2.35)***** |
| P for trend |  | <0.001 | <0.001 |  | <0.001 | <0.001 |

Model 1: adjusting for sex, age, education level, occupation, personal income, smoking status, alcohol consumption, physical activity, support from family members, and contacts with relatives and friends;

Model 2: additionally adjusting for BMI, presence of chronic diseases, and self-rated health;

* P<0.05, **P<0.01, *** P <0.001.

**Supplementary Table 10**. Prevalence and adjusted OR (95% CI) of depressive symptoms by groups of different specific sleep disturbances, stratifying by groups of napping duration

| Variables | Depressive symptoms (%) | Model 1 | Model 2 | Depressive symptoms (%) | Model 1 | Model 2 |
| --- | --- | --- | --- | --- | --- | --- |
| **Non-nappers** | | | | **Napping of <60min** | | |
| Subjective sleep quality |  |  |  |  |  |  |
| Good | 5,606 (3.3) | 1.00 | 1.00 | 2,860 (4.4) | 1.00 | 1.00 |
| Poor | 1,093 (11.4) | **3.42 (2.67, 4.38)***** | **3.37 (2.62, 4.32)***** | 497 (10.7) | **2.55 (1.80, 3.61)***** | **2.43 (1.71, 3.45)***** |
| P |  | <0.001 | <0.001 |  | <0.001 | <0.001 |
| Sleep latency |  |  |  |  |  |  |
| ≤30 min | 5,826 (3.8) | 1.00 | 1.00 | 2,951 (5.0) | 1.00 | 1.00 |
| 31-60 min | 640 (7.8) | **1.82 (1.31, 2.53)***** | **1.78 (1.28, 2.47)***** | 303 (6.3) | 1.15 (0.69, 1.91) | 1.13 (0.67, 1.88) |
| >60 min | 233 (15.5) | **3.39 (2.27, 5.07)***** | **3.37 (2.25, 5.04)***** | 103 (14.6) | **2.93 (1.62, 5.33)***** | **2.83 (1.55, 5.15)**** |
| P for trend |  | <0.001 | <0.001 |  | 0.002 | 0.003 |
| Sleep duration |  |  |  |  |  |  |
| <5 h/day | 393 (12.2) | **3.13 (2.15, 4.57)***** | **3.16 (2.17, 4.61)***** | 187 (8.0) | 1.46 (0.80, 2.67) | 1.37 (0.75, 2.52) |
| 5-6 h/day | 2,525 (5.7) | **1.59 (1.22, 2.07)**** | **1.58 (1.21, 2.06)**** | 1,335 (6.4) | 1.35 (0.96, 1.89) | 1.38 (0.99, 1.94) |
| 7-8 h/day | 3,161 (3.3) | 1.00 | 1.00 | 1,550 (4.3) | 1.00 | 1.00 |
| >8 h/day | 379 (1.9) | 0.55 (0.25, 1.21) | 0.54 (0.25, 1.19) | 138 (2.9) | 0.71 (0.25, 2.02) | 0.70 (0.25, 1.98) |
| P for trend |  | 0.026 | 0.030 |  | 0.201 | 0.174 |
| Sleep efficiency |  |  |  |  |  |  |
| ≥85% | 4,551 (3.4) | 1.00 | 1.00 |  | 1.00 | 1.00 |
| 75-84% | 1,028 (5.7) | **1.49 (1.09, 2.05)*** | **1.47 (1.07, 2.02)***** | 2,231 (4.6) | 1.07 (0.70, 1.63) | 1.10 (0.72, 1.69) |
| 65-74% | 564 (8.7) | **2.33 (1.65, 3.30)***** | **2.30 (1.63, 3.26)***** | 568 (5.5) | 1.55 (0.93, 2.56) | 1.50 (0.90, 2.44) |
| <65% | 556 (8.6) | **2.25 (1.58, 3.19)***** | **2.23 (1.57, 3.16)***** | 269 (7.8) | 1.59 (0.99, 2.55) | 1.52 (0.95, 2.44) |
| P for trend |  | <0.001 | <0.001 |  | 0.023 | 0.038 |
| **Napping of 60-90 min** | | | | **Napping of >90 min** | | |
| Subjective sleep quality |  |  |  |  |  |  |
| Good | 4,807 (4.3) | 1.00 | 1.00 | 938 (6.2) | 1.00 | 1.00 |
| Poor | 821 (12.8) | **2.99 (2.31, 3.87)***** | **2.97 (2.30, 3.85)***** | 164 (18.3) | **3.29 (1.98, 5.48)***** | **3.23 (1.93, 5.40)***** |
| P |  | <0.001 | <0.001 |  | <0.001 | <0.001 |
| Sleep latency |  |  |  |  |  |  |
| ≤30 min | 4,836 (4.7) | 1.00 | 1.00 | 929 (6.7) | 1.00 | 1.00 |
| 31-60 min | 603 (9.6) | **1.85 (1.35, 2.53)***** | **1.84 (1.35, 2.53)***** | 124 (13.7) | **2.04 (1.11, 3.75)*** | **2.01 (1.09, 3.71)*** |
| >60 min | 189 (12.7) | **2.66 (1.67, 4.24)***** | **2.67 (1.67, 4.25)***** | 49 (18.4) | **3.15 (1.39, 7.15)**** | **3.08 (1.35, 7.03)**** |
| P for trend |  | <0.001 | <0.001 |  | 0.001 | 0.001 |
| Sleep duration |  |  |  |  |  |  |
| <5 h/day | 245 (9.4) | **1.80 (1.11, 2.92)*** | **1.78 (1.09, 2.88)*** | 68 (17.7) | **3.32 (1.51, 7.26)**** | **3.10 (1.41, 6.84)**** |
| 5-6 h/day | 2,224 (7.4) | **1.64 (1.28, 2.11)***** | **1.65 (1.28, 2.11)***** | 418 (10.3) | **2.05 (1.22, 3.44)**** | **2.07 (1.23, 3.48)**** |
| 7-8 h/day | 2,690 (4.3) | 1.00 | 1.00 | 515 (5.2) | 1.00 | 1.00 |
| >8 h/day | 257 (2.7) | 0.58 (0.27, 1.28) | 0.58 (0.26, 1.26) | 69 (7.3) | 1.27 (0.46, 3.54) | 1.29 (0.46, 3.58) |
| P for trend |  | 0.007 | 0.007 |  | 0.036 | 0.033 |
| Sleep efficiency |  |  |  |  |  |  |
| ≥85% | 3,814 (4.2) | 1.00 | 1.00 | 750 (5.9) | 1.00 | 1.00 |
| 75-84% | 889 (8.0) | **1.83 (1.36, 2.47)***** | **1.84 (1.37, 2.48)***** | 179 (12.3) | **2.06 (1.17, 3.63)*** | **2.10 (1.19, 3.70)*** |
| 65-74% | 488 (7.2) | **1.61 (1.09, 2.38)*** | **1.60 (1.09, 2.36)*** | 82 (14.6) | **2.83 (1.38, 5.79)**** | **2.71 (1.32, 5.58)**** |
| <65% | 437 (10.1) | **2.16 (1.51, 3.10)***** | **2.11 (1.47, 3.03)***** | 91 (11.0) | 1.33 (0.61, 2.88) | 1.25 (0.57, 2.73) |
| P for trend |  | <0.001 | <0.001 |  | 0.023 | 0.040 |

Model 1: adjusting for sex, age, education level, occupation, personal income, smoking status, alcohol consumption, physical activity, support from family members, and contacts with relatives and friends;

Model 2: additionally adjusting for BMI, presence of chronic diseases, and self-rated health;

* P<0.05, **P<0.01, *** P <0.001.

**Supplementary Table 11**. Prevalence and adjusted OR (95% CI) of depressive symptoms by groups of different specific sleep disturbances, stratifying by groups of napping frequency

| Variables | Depressive symptoms (%) | Model 1 | Model 2 | Depressive symptoms (%) | Model 1 | Model 2 |
| --- | --- | --- | --- | --- | --- | --- |
| **Non-nappers** | | | | **Napping of 1-3 days/week** | | |
| Subjective sleep quality |  |  |  |  |  |  |
| Good | 5,606 (3.3) | 1.00 | 1.00 | 1,970 (3.7) | 1.00 | 1.00 |
| Poor | 1,093 (11.4) | **3.42 (2.67, 4.38)***** | **3.37 (2.62, 4.32)***** | 336 (11.0) | **3.02 (1.96, 4.67)***** | **2.91 (1.87, 4.51)***** |
| P |  | <0.001 | <0.001 |  | <0.001 | <0.001 |
| Sleep latency |  |  |  |  |  |  |
| ≤30 min | 5,826 (3.8) | 1.00 | 1.00 | 2,021 (4.2) | 1.00 | 1.00 |
| 31-60 min | 640 (7.8) | **1.82 (1.31, 2.53)***** | **1.78 (1.28, 2.47)***** | 220 (8.6) | **1.92 (1.12, 3.27)*** | **1.80 (1.05, 3.09)*** |
| >60 min | 233 (15.5) | **3.39 (2.27, 5.07)***** | **3.37 (2.25, 5.04)***** | 65 (9.2) | 2.32 (0.95, 5.67) | 2.20 (0.88, 5.47) |
| P for trend |  | <0.001 | <0.001 |  | 0.003 | 0.007 |
| Sleep duration |  |  |  |  |  |  |
| <5 h/day | 393 (12.2) | **3.13 (2.15, 4.57)***** | **3.16 (2.17, 4.61)***** | 107 (7.5) | 1.60 (0.70, 3.63) | 1.66 (0.73, 3.77) |
| 5-6 h/day | 2,525 (5.7) | **1.59 (1.22, 2.07)**** | **1.58 (1.21, 2.06)**** | 914 (6.5) | **1.60 (1.05, 2.44)*** | **1.57 (1.03, 2.39)*** |
| 7-8 h/day | 3,161 (3.3) | 1.00 | 1.00 | 1,100 (3.7) | 1.00 | 1.00 |
| >8 h/day | 379 (1.9) | 0.55 (0.25, 1.21) | 0.54 (0.25, 1.19) | 102 (2.0) | 0.48 (0.11, 2.09) | 0.47 (0.11, 2.02) |
| P for trend |  | 0.026 | 0.030 |  | 0.133 | 0.189 |
| Sleep efficiency |  |  |  |  |  |  |
| ≥85% | 4,551 (3.4) | 1.00 | 1.00 | 1,567 (4.1) | 1.00 | 1.00 |
| 75-84% | 1,028 (5.7) | **1.49 (1.09, 2.05)*** | **1.47 (1.07, 2.02)***** | 354 (5.9) | 1.35 (0.80, 2.27) | 1.31 (0.77, 2.21) |
| 65-74% | 564 (8.7) | **2.33 (1.65, 3.30)***** | **2.30 (1.63, 3.26)***** | 204 (7.4) | 1.65 (0.90, 3.01) | 1.47 (0.80, 2.71) |
| <65% | 556 (8.6) | **2.25 (1.58, 3.19)***** | **2.23 (1.57, 3.16)***** | 181 (5.5) | 1.10 (0.54, 2.24) | 1.06 (0.52, 2.18) |
| P for trend |  | <0.001 | <0.001 |  | 0.275 | 0.420 |
| **Napping of 4-6 days/week** | | | | **Daily napping** | | |
| Subjective sleep quality |  |  |  |  |  |  |
| Good | 1,295 (4.1) | 1.00 | 1.00 | 5,340 (5.0) | 1.00 | 1.00 |
| Poor | 205 (7.8) | **2.04 (1.09, 3.80)*** | **2.06 (1.10, 3.84)*** | 941 (14.4) | **2.97 (2.36, 3.73)***** | **2.90 (2.30, 3.64)***** |
| P |  | 0.025 | 0.024 |  | <0.001 | <0.001 |
| Sleep latency |  |  |  |  |  |  |
| ≤30 min | 1,295 (3.9) | 1.00 | 1.00 | 5,400 (5.6) | 1.00 | 1.00 |
| 31-60 min | 162 (6.2) | 1.40 (0.66, 2.97) | 1.43 (0.68, 3.04) | 648 (10.0) | **1.63 (1.21, 2.18)**** | **1.61 (1.20, 2.16)**** |
| >60 min | 43 (18.6) | **7.41 (3.02, 18.20)***** | **7.47 (3.01, 18.52)***** | 233 (14.6) | **2.55 (1.72, 3.79)***** | **2.51 (1.69, 3.73)***** |
| P for trend |  | <0.001 | <0.001 |  | <0.001 | <0.001 |
| Sleep duration |  |  |  |  |  |  |
| <5 h/day | 62 (8.1) | **3.09 (1.04, 9.20)*** | **3.04 (1.02, 9.05)*** | 331 (11.2) | **1.80 (1.22, 2.67)**** | **1.74 (1.17, 2.58)**** |
| 5-6 h/day | 521 (8.3) | **3.22 (1.79, 5.80)***** | **3.21 (1.78, 5.80)***** | 2,542 (7.5) | **1.38 (1.10, 1.72)**** | **1.38 (1.10, 1.73)**** |
| 7-8 h/day | 739 (2.4) | 1.00 | 1.00 | 2,916 (5.2) | 1.00 | 1.00 |
| >8 h/day | 93 (3.2) | 1.41 (0.39, 5.09) | 1.39 (0.38, 5.03) | 269 (4.1) | 0.76 (0.40, 1.42) | 0.74 (0.39, 1.40) |
| P for trend |  | 0.001 | 0.001 |  | 0.062 | 0.059 |
| Sleep efficiency |  |  |  |  |  |  |
| ≥85% | 1,051 (3.3) | 1.00 | 1.00 | 4,177 (5.0) | 1.00 | 1.00 |
| 75-84% | 238 (6.3) | 1.82 (0.94, 3.51) | 1.82 (0.94, 3.52) | 1,044 (8.4) | **1.58 (1.21, 2.06)**** | **1.61 (1.23, 2.10)***** |
| 65-74% | 114 (9.7) | **3.10 (1.46, 6.60)**** | **3.09 (1.45, 6.60)**** | 521 (8.1) | **1.50 (1.05, 2.14)*** | **1.49 (1.04, 2.12)*** |
| <65% | 97 (8.3) | 2.29 (0.98, 5.37) | 2.33 (0.99, 5.49) | 539 (11.5) | **1.98 (1.45, 2.70)***** | **1.93 (1.41, 2.63)***** |
| P for trend |  | 0.002 | 0.001 |  | <0.001 | <0.001 |

Model 1: adjusting for sex, age, education level, occupation, personal income, smoking status, alcohol consumption, physical activity, support from family members, and contacts with relatives and friends;

Model 2: additionally adjusting for BMI, presence of chronic diseases, and self-rated health;

* P<0.05, **P<0.01, *** P <0.001.
